# Risky sexual behavior practice and associated factors among secondary and preparatory school students of Aksum town, northern Ethiopia, 2018

**DOI:** 10.1186/s13104-019-4714-1

**Published:** 2019-10-26

**Authors:** Alem Girmay, Teklewoini Mariye

**Affiliations:** School of Nursing, College of Health Science, Aksum University, Aksum, Ethiopia

**Keywords:** Risk sexual behavior, High school student, Cross sectional study, Aksum

## Abstract

**Objective:**

As adolescence is a stage in which human beings face once throughout a lifetime and it is the experimental period for this reason risky sexual behavior is common among young people, and it has several sexual and reproductive health consequences. But it doesn’t get enough attention the status of risky sexual behavior practice and factors’ contributing to it, so this study aimed to identify the prevalence of risky sexual behavior and factors associated with it.

**Results:**

From the total respondents 115 (23.7%) had history of sexual intercourse and the mean age for sexual initiation is 15.48 ± 1.99 year. From whom 110 (98.2%) had sexual contact below the age of 18 and only 68 (60.8%) initiate first sex by their own willing. Overall 97 (19.6%) had practiced risky sexual behaviors. Students not facing peer pressure were 0.36 times less likely to develop risk sexual behavior (AOR = 0.357, 95% CI 0.172, 0.744).

## Introduction

According to different sources the term youth refers to the age interval in between 15 and 24 years old and Since peoples become sexual active in this age interval healthy sexual awareness and development is mandatory for the future health status of the youths and adolescents in particular [1, 2]. Healthy sexual development contributes for holistic personal well-being but if youths and adolescents become unaware of this they will develop different risky sexual behaviors [2–6].

Risky sexual behavior affects the youths and adolescents life style and contributes to different adverse effects, but as reports indicating its prevalence is increasing. One report indicates 41% adolescents had ever had sexual contact; from this number 43% didn’t use any protective including condom the last time they had sex, 14% did not use any contraceptive, 21% had drunk alcohol before sexual intercourse [4, 7].

According to different studies adolescents are at high risk of developing risky sexual behavior with different environmental and communicational factors, for this case the global prevalence of HIV/AIDS which directly correlates with risk sexual behavior is increasing, sub Saharan countries are the most affected for this including Ethiopia with a prevalence of age 15–19 years (0.2%) and 20–24 years (0.9%) [5, 8].

As the study in China the prevalence of risk sexual behavior was 42.4%, even in Ethiopia risk sexual behavior among secondary school students was relatively high, for instance it was reported in Addis Ababa 26.7% and especially in Debre-brhan, 6.7% of youths practiced sex with commercial sex workers [5, 9, 10].

Despite of health policy maker’s effort to create awareness and to reduce sexually transmitted infections (STI), contracting HIV/STIs is at an increasing rate. Since High school students are mainly in age group of 15–24, they are more exposed to risky sexual behavior [9].

## Main text

### Study setting and period

The study was conducted from March 18 to 25/2018 in Aksum town secondary and preparatory schools, which is located at 1024 km away from Addis Ababa. The total number of younger population in the town is estimated to be 44,260 out of which 24,292 of the total population is considered as adolescent.

### Study design, population and eligibility criteria

Institutional based quantitative study design was applied. All systematically selected students from those registered for grade 9–12 and consented/assented were included in the study.

### Sample size calculation and Sampling Procedure

The sample size was calculated using a single population proportion formula by considering the proportion of risky sexual practice as 71.2% [10] and 5% margin of error, Correction formula since the total population was less than 10,000 which was 6939 and 1.5 design effect, sample size was calculated for different significant variables and finally 498 was obtained. Sample size has been allocated for every grade based on proportional allocation to their size. Finally students from every class had been selected by systematic random sampling (k = 14).

### Data collection and analysis process

Data were collected using a standardized and pre-tested self-administered questionnaire adapted from WHO sexual and Reproductive Health [11]. Experienced supervisors and data collectors were selected and trained prior to the survey. Completeness of each questionnaire had been checked. Double data entry was done and consistency of the entered data was cross checked by comparing the two separately entered data on Epi Data. Data were cleaned and entered to Epi Data version 3.0.2 and exported to SPSS version 22 for further analysis. Frequency, percentage, means and standard deviation was used for data description. Bivariate logistic regression was applied to identify associated factors then those with p-value ≤ 0.25 were entered to multivariate logistic regression to get the significantly associated variables with p-value < 0.05.

### Variables, operational definitions and ethical issues

The dependent variable was risky sexual behavior and the independent variables were socio demographic characteristics, Parental characteristics and other relevant data. The operational definition for risky sexual behavior was when a student practices at least one of the following, multiple sexual partners (having more than one sexual partner until the survey), early initiation of sex (sexual debut at the age < 18 years old), inconsistent use of condom (inconsistent/fail to use condom at least ones during sexual intercourse until the survey), Sex with commercial sex workers at least once until the survey).

Ethical clearance had been obtained from Institutional review committee of Aksum University college of Health Science. Permission to conduct the research was obtained from local government authorities and school directors. Data were collected after full informed written consent/assent was obtained from each study subject’s accordingly. When age was less than 16 years old, it obtained from parents/legal guardians of students. Confidentiality was secured and treated anonymous.

## Results

### Socio demographic characteristics of study participants

A total of 496 students completed the questionnaire yielding a total response rate of 99.6%. Among those respondents, the large proportion 253 (51.1%) were females by gender. The mean age of participants was 16.29 years (± 1.45), ranging from 13 to 24 years old. Among the respondents 68.1% of the students were living with their parents and 405 (82%) are raised by both parents (Table 1).

**Table 1:** Socio-demographic characteristics of secondary and preparatory school students of Aksum town, northern Ethiopia, 2018 (n = 496)

Variable (n = 496)	Frequency (%)
Age	
14–19	479 (96.6)
20–24	15 (3.4)
Sex	
Male	242 (48.9)
Female	253 (51.1)
Level of education	
Grade 9	203 (41.0)
Grade 10	228 (46.1)
Grade 11	44 (8.90)
Grade 12	20 (4.0)
Religion	
Orthodox	461 (93.1)
Muslim	25 (5.1)
Protestant	8 (1.6)
Catholic	1 (0.2)
Ethnicity	
Tigray	437 (88.3)
Amhara	39 (7.9)
Oromo	19 (3.8)
Raised by	
Both parents	405 (82)
With father/mother	13 (2.6)
Other relatives	76 (15.4)
Currently live with	
Both parents	337 (68.1)
Father	24 (4.8)
Mother	77 (15.6)
Alone	48 (9.7)
Others	9 (1.8)

### Prevalence of risk sexual behavior

One out of four 115 (23.7%) of the students had ever practiced sexual intercourse and the mean age for sexual initiation was 15.48 year (± 1.99). 110 (98.2%) had sexual contact below the age of 18, 30 (26.3%) had ≥ 2 life time sexual partner and 44 (29.3%) had ≥ 2 sexual partners in the last 12 months. Moreover 54 (46.6%) hasn’t been use condom consistently and 41 (35%) had faced objection for condom use by their sexual partner. Overall 97 (19.6%) had practiced risky sexual behaviors (Table 2).

**Table 2:** Parental and other related characteristics of secondary and preparatory school students of Aksum town, northern Ethiopia, 2018 (n = 496)

Variable (n = 496)	Frequency (%)
Father currently alive	
Yes	444 (89.7)
No	51 (10.3)
Father’s educational status	
Can’t read and write	38 (7.7)
Read and write	235 (47.6)
Elementary	139 (28.1)
High school College and above	82 (16.6)
Father’s employment status	
Employed	247 (50.3)
Unemployed	244 (47.7)
Mother currently alive	
Yes	466 (94.1)
No	28 (5.9)
Mother’s educational status	
Can’t read and write	104 (21.1)
Read and write	212 (42.9)
Elementary	114 (23.1)
High school college and above	64 (13.0)
Mother’s employment status	
Employed	165 (33.5)
Unemployed	328 (66.5)
Parents control students	
Yes	464 (93.7)
No	31 (6.3)
Discuss on SRH issues with parents	
Yes	377 (76.3)
No	117 (24.7)
Have you ever consumed alcohol	
Yes	114 (23.0)
NO	381 (77.0)
Have you ever smoke cigarette	
Yes	23 (4.6)
No	472 (95.4)
Pocket money per month	
1–150 birr	349 (70.5)
151–250 birr	62 (12.5)
251–500 birr	30 (6.1)
> 500 birr	54 (10.9)
Peer pressure	
Yes	59 (11.9)
No	435 (88.1)
Ever seen/read pornography	
Yes	160 (32.3)
No	335 (67.7)
Ever had sexual intercourse	
Yes	115 (23.7)
No	376 (76.6)
Age at first sex (n = 115)	
< 18 years	110 (95.6)
> 18 years	5 (4.4)
Relationship of first sexual partner	
Boy/girlfriend	84 (73.7)
Teacher	30 (25.3)
Others	1 (1)
Initiations of first sex	
Own will	68 (60.8)
Forced	18 (16.0)
For money	10 (9.0)
Materials/gifts	16 (14.2)
Lifetime sexual partners	
One	84 (73.7)
Two and more	30 (26.3)
Sexual partners in the last 12 month	
One	68 (60.7)
Two and more	44 (29.3)
Ever had sex for cash or gift	
Yes	39 (32)
No	83 (68)
Condom used for first sex	
Yes	65 (55.6)
No	52 (44.4)
Suggestion for condom use	
Myself	45 (39.8)
My partner	24 (21.2)
Joint	44 (38.9)
Use condom at last sexual intercourse	
Yes	56 (48.7)
No	59 (51.3)
Consistence condom use	
Yes	62 (53.4)
No	54 (46.6)
Partner’s objection on condom use	
Yes	41 (35.0)
No	76 (65.0)

### Factors associated with risk sexual behavior

In the multivariate logistic regression by whom raised the student, discussion on SRH with parents, sex with Commercial sex workers, pocket money, peer pressure and pornographies had significant association with risky sexual behavior (Table 3).

**Table 3:** Factors associated with risk sexual behaviors of secondary and preparatory school students of Aksum town, northern Ethiopia, 2018 (n = 496)

Variables	Risk sexual behaviors		P-value	COR (95% CI)	P-value	AOR (95% CI)
	Yes	No				
Raised by						
Both parents	83	322	0.035	0.56 (0.33, 0.96)	0.42	0.46 (0.2, 0.9)
With father/mother	4	9	0.95	0.96 (0.27, 3.4)	0.31	2.2(0.47, 10.8)
Other relatives	24	52	1	1	1	1
Discussion on SRH						
Yes	96	281	1	1	1	1
No	15	102	0.005	2.3 (1.3, 4.2)	0.001	3.42 (1.6, 7.3)
Sex with CSWs						
Yes	23	3	0.001	33.2 (9.7, 113)	0.001	18 (4.0, 75.4)
No	88	381	1	1	1	1
Monthly pocket money						
1–150 birr	65	287	0.01	0.29 (0.16, 0.5)	0.02	0.34 (0.13, 0.84)
151–250 birr	15	47	0.037	0.43 (0.195, 0.95)	0.043	0.31 (0.1, 0.96)
251–500 birr	11	19	0.596	0.78 (0.3, 1.95)	0.86	1.2 (0.3, 3.9)
> 500 birr	23	31	1	1	1	1
Peer pressure						
Yes	34	26	0.001	0.12 (0.09, 0.3)	0.007	0.36 (0.17, 0.74)
No	77	358	1	1	1	1
Seen pornographic movies						
Yes	75	85	0.001	7.3 (4.6, 11.7)	0.001	5.9 (3, 11.47)
No	36	299	1	1	1	1

## Discussion

In Ethiopia, many studies have been conducted on risk sexual behavior in different population groups, however the issue was neglected among young secondary school students in the study area. Thus, this study was able to assess the prevalence of risk sexual behavior and associated factors among high school students in Aksum town.

From the surveyed students, 23.7% had ever practiced sexual intercourse and the mean age for sexual initiation was 15.48 year (± 1.99), which is higher than the studies finding in China (7%) [12], (13.8%) India, (18.3%) Aleta wondo, (19%) Shendi, and comparable with the study finding in North Gondar (23%), lower than the studies finding in Colombia (25%), (27.1%) Gurage, (29%) Wolaita, Bodetti, south Ethiopia (29.1%), Assela (33.6%), and South Africa (47%) [9, 12–21]. The disparity might be due to difference in study participant Sociodemographic difference, difference in traditional and cultural background, and difference.

The prevalence of risk sexual behavior practice in this study is 19.6%, which is comparable with study finding in assela 20% [20], northwest Ethiopia 20.4% [22], higher than the studies finding in Addis Ababa by Amsale Cherie et al. (10.6%) [23], Humera (13.7%) [24], Jijiga (16%) [25], Bodetti, southern Ethiopia (17.9%) [26], and lower than the studies finding in Nigeria (24.1%) [27], Benishangul Gumuz (82.2%) [28], Haramaya (65.8%) [29], Addis Ababa by Abdusemed Mussa Ali (71.6%) [30], (48.3%) Dilla town [31], (70.5%) Agena Guragae zone, Ethiopia [13] and (42.1%) China [12]. This difference might be due to the approaches taken to quantify risky sexual behavior, study area variation and study participants Sociodemographic characteristics.

Those students not discussed on sexual reproductive health with parents are 3.4 times more likely to practice risky sexual behavior. In line with the finding in Assela [20], Dilla town [31], Haromaya [32]; Benishangul Gumuz [28]; Metema [33], and in contrast to studies finding in bale goba [34], and Nepal [35]. This might be since majority of student preferred to discuss about sexual issues with their peers of the same sex than with their family members, even if they discussed with their family they may not give attention for the discussion or the discussion topics are limited and not to the satisfaction level of the students, as a result they may develop immature awareness, which contributes to the practice.

Students having sex with commercial sex workers are eighteen times more likely to practice risky sexual behavior. It is consistent with study finding in Benishangul Gumuz [28] but studies in Humera [24], Jimma [36], Haramaya [29] not supported the evidence. This might be since those CSWs have relation with many people, again many of them gives more attention for money than safety that means they are at high sexual risk, as adolescents have sexual relation with those people they are vulnerable for risky sexual behavior practice [24].

Respondents seeing pornographies are 6 times more likely practices risky sexual behavior. This is similar with the study finding in Jimma [36], Addis Ababa [30], Haramaya [32] and Humera [24] but in contrast in other study finding in Addis Ababa done by Gizaw et al. [10], and in Haramaya [37]. The possible reason for the discrepancy might be since some students may get experience on how to prevent risky sexual practice whereas other groups may be liable and need to enjoy what they observe in the film without any protection.

Students not facing peer pressure are 64% less likely practices risky sexual behavior. This is in line with study finding in Slovak [38], South Africa [39], Ghana [40], Addis Ababa [23], Assela [20] and humera [22], this might be due to their need to share life experiences with their peers, however, study in Addis Ababa by Gizaw et al. [10] revealed that peer pressure had no statistical significant effect, which might be due to self-efficacy of students to wards external force.

Students who got < 150 Birr monthly pocket money had 66% less likely practices risky sexual behavior than those students who got monthly pocket money greater than 500 Birr. This might be due to the reason that when adolescents get money they may have opportunity to practice what they desire and the adolescents age interval demands high sexual desire, it is in contrast with the study finding in Bodetti, south Ethiopia which reports those didn’t get money 1.2 times more likely to practice risky sexual behavior.

Respondents who rose by both parents had 54% less likely practices risky sexual behavior than those who rose by either of the parents (AOR = 0.46 (0.2, 0.9). supported by findings in Slovakia [38], Sweden [41], Humera [22], Jimma [36], benishangul Gumuz [42], western Ethiopia [43], African countries [44], Haromaya [29].

## Conclusions

In this study the prevalence of risky sexual behavior reported as 19.6%, discussion on SRH with parents, raised by both parents and < 150 birr monthly pocket money had preventive significant association. Peer pressure, seeing pornographies and sex with CSWs had significant association with increasing risk sexual behavior.

## Limitations

Since the topic is sensitive issue there may be over reporting or under reporting and due to the nature of the study design may not show cause and effect so that it should be repeated with different study design.

## Data Availability

The data sets used and/or analyzed during the current study available from the corresponding author on reasonable request.

## Abbreviations

CI: confidence interval
AOR: adjusted odd ratio
SPSS: Statistical Package for Social Sciences
